# Impact of metal artifact reduction algorithm on gross tumor volume delineation in tonsillar cancer: reducing the interobserver variation

**DOI:** 10.1186/s12880-022-00889-0

**Published:** 2022-09-06

**Authors:** Yoshiyuki Fukugawa, Ryo Toya, Tomohiko Matsuyama, Takahiro Watakabe, Yoshinobu Shimohigashi, Yudai Kai, Tadashi Matsumoto, Natsuo Oya

**Affiliations:** 1 Department of Radiation Oncology, Faculty of Life Sciences, Kumamoto University, 1-1-1 Honjo, Chuo-ku, Kumamoto, 860-8556 Japan; 2 Department of Radiological Technology, Kumamoto University Hospital, 1-1-1 Honjo, Chuo-ku, Kumamoto, 860-8556 Japan

**Keywords:** Metal artifact reduction, Radiotherapy, Head and neck cancer, Oropharyngeal carcinoma, Radiotherapy planning, Gross tumor volume

## Abstract

**Background:**

Patients with tonsillar cancer (TC) often have dental fillings that can significantly degrade the quality of computed tomography (CT) simulator images due to metal artifacts. We evaluated whether the use of the metal artifact reduction (MAR) algorithm reduced the interobserver variation in delineating gross tumor volume (GTV) of TC.

**Methods:**

Eighteen patients with TC with dental fillings were enrolled in this study. Contrast-enhanced CT simulator images were reconstructed using the conventional (CT_CONV_) and MAR algorithm (CT_MAR_). Four board-certified radiation oncologists delineated the GTV of primary tumors using routine clinical data first on CT_CONV_ image datasets (GTV_CONV_), followed by CT_CONV_ and CT_MAR_ fused image datasets (GTV_MAR_) at least 2 weeks apart. Intermodality differences in GTV values and Dice similarity coefficient (DSC) were compared using Wilcoxon’s signed-rank test.

**Results:**

GTV_MAR_ was significantly smaller than GTV_CONV_ for three observers. The other observer showed no significant difference between GTV_CONV_ and GTV_MAR_ values. For all four observers, the mean GTV_CONV_ and GTV_MAR_ values were 14.0 (standard deviation [SD]: 7.4) cm^3^ and 12.1 (SD: 6.4) cm^3^, respectively, with the latter significantly lower than the former (*p* < 0.001). The mean DSC of GTV_CONV_ and GTV_MAR_ was 0.74 (SD: 0.10) and 0.77 (SD: 0.10), respectively, with the latter significantly higher than that of the former (*p* < 0.001).

**Conclusions:**

The use of the MAR algorithm led to the delineation of smaller GTVs and reduced interobserver variations in delineating GTV of the primary tumors in patients with TC.

## Background

Radiotherapy (RT) with or without chemotherapy is an organ preservation therapy commonly indicated for tonsillar cancer (TC) [1]. High-precision RT techniques such as intensity-modulated radiotherapy and volumetric-modulated arc therapy have become increasingly used for the treatment of TC [2]. As these RT techniques are characterized by highly conformal dose distributions, the treatment success highly depends on the accurate definition of gross tumor volume (GTV). Delineating GTV based on computed tomography (CT) simulator images is required for the RT planning process for TC. However, patients with TC often have dental fillings with metal artifacts in CT scans. Metal artifacts can significantly degrade the quality of simulation CT images, obscure visualization of the primary tumor, and therefore result in a large degree of interobserver variations in delineating GTV of primary tumors [3].

Recently, metal artifact reduction (MAR) algorithms have been increasingly used for CT imaging for the diagnosis and RT planning of head and neck cancer [4–7]. The commercially available software Smart MAR (GE Healthcare, Chicago, IL, USA) reduces photon starvation, beam hardening, and streak artifacts caused by high z metals in the body [5]. However, the usefulness of MAR algorithms in delineating the GTV of patients with TC is not fully discussed. Therefore, this study aimed to evaluate whether the use of MAR algorithms reduces interobserver variations in delineating the GTV of TC.

## Methods

### Patients

This retrospective study was approved by the institutional review board of our hospital. Between July 2019 and August 2021, 21 patients with pathologically confirmed tonsillar squamous cell carcinoma with dental fillings underwent pretreatment contrast-enhanced MR imaging within 4 weeks and contrast-enhanced [^18^F]-fluoro-2-deoxy-D-glucose (FDG)–positron emission tomography (PET)/CT within 6 weeks before RT planning contrast-enhanced CT imaging in our hospital [2]. Three patients were excluded due to the presence of superficial T1 lesions [3]. Finally, the study population consisted of 18 patients, comprising 12 males and 6 females (median age 57 [range 48–88] years). Patients were categorized according to the Union for International Cancer Control TNM staging system, 8th edition: 13 patients with T2, 4 with T3, and 1 with T4 tumors. The p16 status was positive in 15 and negative in 3 patients.

### Imaging protocol

The pretreatment imaging protocols were described elsewhere [2, 8]. RT planning CT images with iodinated contrast media were acquired with a 16-row CT scanner (Discovery RT; GE Healthcare, Chicago, IL, USA). During CT scanning, patients were positioned supine with arms by their sides. A pillow and a thermoplastic mask dedicated to RT were used [9, 10]. A total of 80–100 ml of contrast media (Omnipaque 300, Daiichi-Sankyo, Tokyo, Japan) was injected at a 2 ml/s rate using a 22-G intravenous catheter placed in an antecubital vein. The CT scan was conducted 50–60 s after the initiation of contrast injection with helical mode, 120 kVp, Smart mA automatic exposure control (GE Healthcare, Chicago, IL, USA), 2.5-mm slice thickness, and 650-mm field of view (FOV). CT images were reconstructed using 500-mm FOV using the conventional (CT_CONV_) and Smart MAR algorithms (CT_MAR_). CT_MAR_ removed metal artifacts based on the following steps: (1) identification of corrupted samples in the projection that corresponds to metallic objects, (2) generating inpainted data by replacing metal corrupted projections with the corrected data generated using the forward projection of the classified image, and (3) generating the final corrected projection by combining the original and inpainted projection data [11]. CT_CONV_ and CT_MAR_ images were transferred to the RT planning system (Pinnacle^3^ 9.10; Philips Medical Systems, Fitchburg, MA, USA). Registration of images was performed with the RT planning system through hardware arrangement.

### GTV definition

GTVs of primary tumors were delineated independently by four board-certified radiation oncologists with 5–18 years of experience. Observers were provided with routine clinical data (i.e., contrast-enhanced FDG/PET-CT and contrast-enhanced MR images and endoscopy videos) and asked to contour the GTV of each primary tumor on axial slices of CT_CONV_ datasets. For each case, observers defined the GTV first on CT_CONV_ image datasets (GTV_CONV_), followed by CT_CONV_ and CT_MAR_ fused image datasets (GTV_MAR_) at least 2 weeks apart to minimize memory bias and fatigue [9, 12]. When delineating GTV_CONV_, observers could not view CT_MAR_ images. When delineating CT_MAR_, observers could view both CT_CONV_ and CT_MAR_ images. Observers could adjust the window level and width on their preferences for delineating GTV.

### Evaluation of intermodality differences and observer variations

For each of 18 cases, 8 sets of GTVs were delineated: 4 (observers) × 2 (modalities). Analyses were performed based on three-dimensional volume. GTV_CONV_ and GTV_MAR_ values defined by 4 observers were calculated to evaluate intermodality (CT_CONV_ images vs. CT_CONV_ and CT_MAR_ fused images) differences in GTVs. For geometric interobserver comparisons, the Dice similarity coefficient (DSC), which is used to measure the similarity between two samples, was calculated using the following equation:

$${\text{DSC}} = 2 \times {\text{A}} \cap {\text{B}}/\left( {{\text{A}} + {\text{B}}} \right)$$

where A ∩ B is the volume of the intersection between two GTVs of A and B. The DSC ranged from 0 (no overlap) to 1 (perfect match) [10, 13, 14]. The DSC was calculated as the mean DSC of all possible pair combinations for both GTV_CONV_ and GTV_MAR_ [15].

### Statistical analysis

Intermodality differences in GTV values and DSCs were compared using Wilcoxon’s signed-rank test. Statistical calculations were performed using the SPSS software version 25.0 (IBM, Armonk, NY, USA). Differences with *p* values of < 0.05 were considered statistically significant.

## Results

The mean GTV_CONV_ and GTV_MAR_ values for four observers are shown in Table 1. GTV_MAR_ was significantly smaller than GTV_CONV_ for three observers. The other observer showed no significant difference between GTV_CONV_ and GTV_MAR_ values. For all four observers, the mean GTV_CONV_ and GTV_MAR_ values were 14.0 cm^3^ (standard deviation [SD]: 7.4) cm^3^ and 12.1 (SD: 6.4) cm^3^, respectively, indicating that GTV_MAR_ was significantly smaller than GTV_CONV_ (*p* < 0.001, Table 1).

**Table 1:** GTV_CONV_ and GTV_MAR_ values for four observers

Observer	GTV	Mean ± SD, cm^3^	Range, cm^3^	*p* Value
A	GTV_CONV_	15.7 ± 8.2	6.0–31.3	0.002
	GTV_MAR_	12.9 ± 6.9	3.7–30.4	
B	GTV_CONV_	16.1 ± 7.6	4.4–29.5	0.006
	GTV_MAR_	13.3 ± 6.5	7.1–26.3	
C	GTV_CONV_	11.4 ± 7.0	3.5–29.8	0.013
	GTV_MAR_	9.9 ± 5.6	3.3–21.1	
D	GTV_CONV_	12.6 ± 6.3	2.7–25.1	0.433
	GTV_MAR_	12.3 ± 6.4	4.2–28.9	
All observers	GTV_CONV_	14.0 ± 7.4	2.7–31.3	< 0.001
	GTV_MAR_	12.1 ± 6.4	3.3–30.4	

*GTV* gross tumor volume, *SD* standard deviation, *GTV*_*CONV*_ gross tumor volume delineated based on conventional CT images, *GTV*_*MAR*_ gross tumor volume delineated based on the combination of conventional and metal artifact reduction CT images

**Fig. 1:**
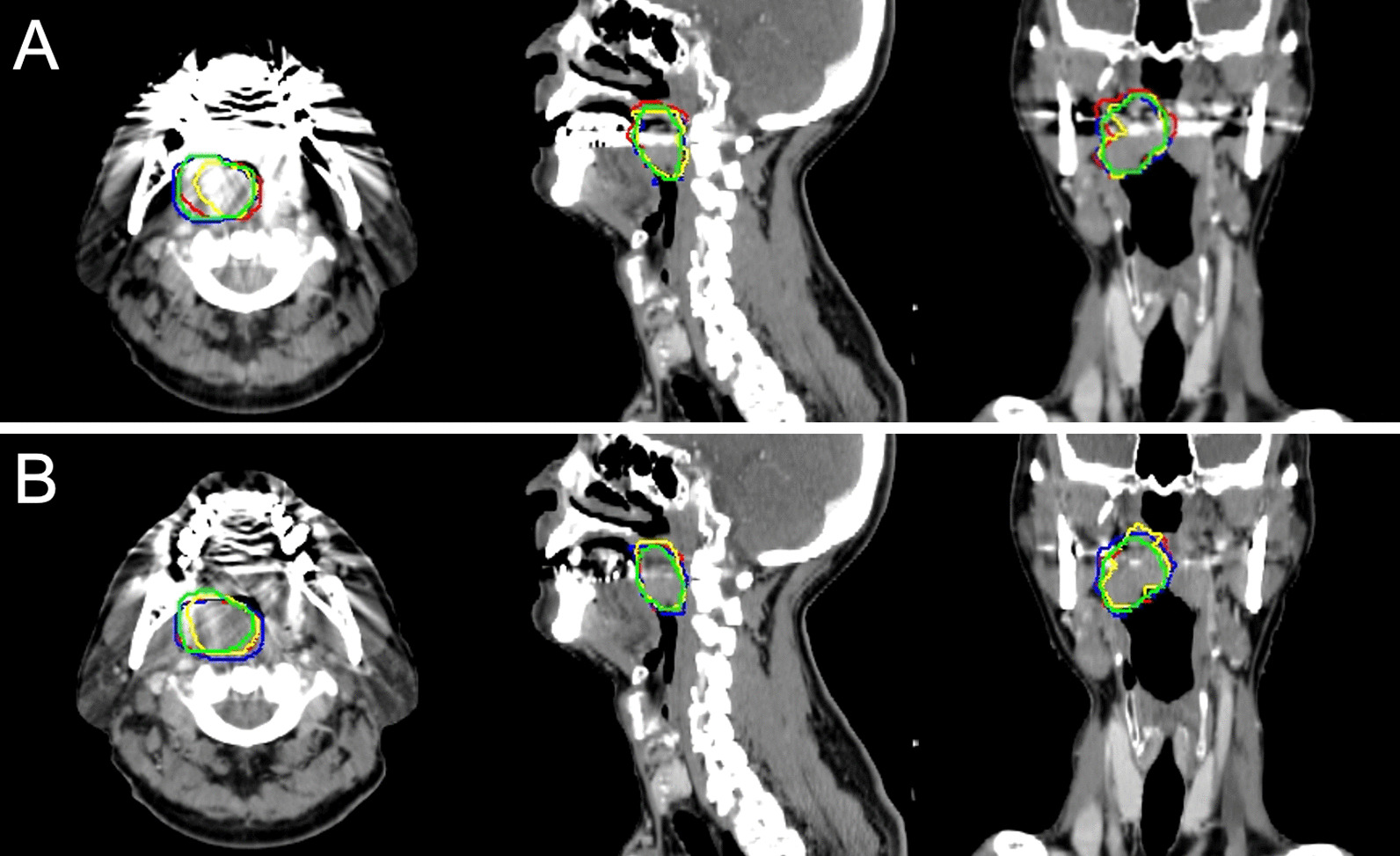
Examples of gross tumor volume (GTV) in patients with tonsillar cancer. Each panel includes axial (left), sagittal (center), and coronal (right) images. **A** GTVs were delineated by four observers using conventional computed tomography (CT) images. The mean Dice similarity coefficient (DSC) was 0.80 with a mean GTV value of 23.7 cm^3^. **B** GTVs were delineated by four observers based on conventional and metal artifact reduction fused CT images. The mean DSC improved to 0.83 with a mean GTV value of 21.6 cm^3^ based on the combination of conventional and metal artifact reduction CT images

The mean DSCs of GTV_CONV_ and GTV_MAR_ were 0.74 (SD: 0.10) and 0.77 (SD: 0.10), respectively, indicating that the DSC of GTV_MAR_ was significantly higher than that of GTV_CONV_ (*p* < 0.001, Table 2). Figure 1 shows a representative patient with DSC of GTV_MAR_ higher than that of GTV_CONV_.

**Table 2:** Dice similarity coefficient of GTV_CONV_ and GTV_MAR_ for each pair and all four observers

Observer	GTV_CONV_ (Mean ± SD)	Range	GTV_MAR_ (Mean ± SD)	Range
A and B	0.71 ± 0.10	0.50–0.82	0.75 ± 0.10	0.49–0.84
A and C	0.76 ± 0.10	0.53–0.89	0.80 ± 0.10	0.63–0.87
A and D	0.75 ± 0.10	0.50–0.86	0.81 ± 0.10	0.48–0.89
B and C	0.71 ± 0.10	0.52–0.84	0.73 ± 0.10	0.51–0.87
B and D	0.75 ± 0.11	0.43–0.87	0.76 ± 0.11	0.47–0.87
C and D	0.76 ± 0.10	0.51–0.86	0.80 ± 0.10	0.58–0.91
All observers	0.74 ± 0.10	0.43–0.89	0.77 ± 0.10	0.47–0.91

*GTV*_*CONV*_ gross tumor volume delineated based on conventional CT images, *SD* standard deviation, *GTV*_*MAR*_ gross tumor volume delineated based on the combination of conventional and metal artifact reduction CT images

## Discussion

RT for TC is associated with acute and late toxicities, including mucositis, dermatitis, taste dysfunction, xerostomia, and osteoradionecrosis. Therefore, an inappropriately large definition of target volumes may lead to deterioration in the quality of life of patients. Our study suggested that the addition of CT_MAR_ to CT_CONV_ images delineated smaller GTVs than CT_CONV_ images alone in patients with TC. One possible reason is that observers unnecessarily included invisible areas due to metal artifacts in GTV_CONV_ images to prevent marginal miss. Abelson et al*.* evaluated the effects of using the MAR technique on GTV delineation in 8 patients with TC [3]. Two radiation oncologists independently delineated the GTV of the primary tumor for each patient based on non-MAR CT (GTV_nonMDT_) and MAR CT (GTV_MDT_) images. GTV_nonMDT_ and GTV_MDT_ values of axial slices with metal artifacts were not significantly different. However, the number of patients was extremely low and may have thus yielded the difference between our results and theirs. The introduction of CT_MAR_ images into RT planning may prevent unnecessary toxicities in patients with TC by reducing target volumes. Conversely, there is a possibility that smaller GTVs yielded using CT_MAR_ images result in inappropriate dose distribution with unintended underdosing to the actual target volume. These benefits and risks should be considered when introducing CT_MAR_ images into RT planning.

Previous studies evaluated the ability of MAR algorithms to improve organ contouring in RT planning. Kohan et al*.* used CT images of 11 patients with metal artifacts in the head and neck regions [16]. Five independent observers with 0–6 years of experience including a medical student performed area measurements of selected normal organ structures, such as masseter muscles and tongues on non-MAR CT and MAR CT image slices with metallic objects and non-MAR CT slices without metallic objects as control. The intraclass correlation coefficient (ICC) was calculated to assess interobserver variations. For all observers, ICCs for non-MAR CT, MAR CT, and control non-MAR CT image slices were 0.903, 0.948, and 0.985 with outliers and 0.884, 0.971, and 0.989 without outliers, respectively. For experienced observers, ICCs for non-MAR CT, MAR CT, and control non-MAR CT image slices were 0.904, 0.979, and 0.976 with outliers and 0.934, 0.975, and 0.990 without outliers, respectively. They suggested the use of MAR algorithms greatly reduced the interobserver variation. Our study results suggested that the addition of CT_MAR_ to CT_CONV_ images reduced interobserver variations compared with CT_CONV_ images alone in delineating GTV of primary tumors in patients with TC. Hansen et al*.* evaluated whether the introduction of MAR algorithms reduces interobserver variations in delineating GTV based on CT images of 11 patients with oropharyngeal cancer (OPC) [13]. Three experienced radiation oncologists and one experienced radiologist independently delineated the GTV of primary tumors (GTV-T) based on non-MAR and MAR CT images. The mean DSCs of GTV-T for non-MAR CT and MAR CT images were 0.60 (SD: 0.24) and 0.61 (SD: 0.20), respectively, showing no difference between the two modalities. The possible reason for the difference between our results and theirs are as follows: (1) the number of their patients was too small and (2) they included patients with OPC other than TC. Greater consistency in delineating GTV with MAR CT images should reduce the influence of potential variability during the RT planning process [17].

Our study has some limitations. First, this was a retrospective study with a relatively small number of patients, although it was larger than previous studies. Second, no pathologic gold standard has been established, which is unavoidable in this type of study. Third, the effects of adding MAR CT images based on dose distributions or treatment outcomes were not evaluated. Further prospective trials based on RT planning simulation using MAR CT images will be required to confirm the clinical benefits for patients with TC.

## Conclusions

The use of MAR CT images in addition to conventional CT images led to the delineation of smaller GTVs and reduced interobserver variations in delineating GTV of primary tumors in patients with TC.

## Data Availability

The data that support the study findings are available from the corresponding author; however, restrictions apply to the data availability, which was used under license for the present study, and thereby are not publicly available. Data are, however, available from the authors upon reasonable request and with permission of the institutional research ethics board of Kumamoto University Hospital.

## Abbreviations

RT: Radiotherapy
TC: Tonsillar cancer
GTV: Gross tumor volume
CT: Computed tomography
MAR: Metal artifact reduction
FDG: [^18^F]-fluoro-2-deoxy-D-glucose
PET: Positron emission tomography
FOV: Field of view
DSC: Dice similarity coefficient
ICC: Intraclass correlation coefficient
